# In diabetic male Wistar rats, quercetin-conjugated superparamagnetic iron oxide nanoparticles have an effect on the SIRT1/p66Shc-mediated pathway related to cognitive impairment

**DOI:** 10.1186/s40360-023-00725-3

**Published:** 2023-12-21

**Authors:** Mahnaz Karami Chamgordani, Akram Bardestani, Shiva Ebrahimpour, Abolghasem Esmaeili

**Affiliations:** Department of Cell and Molecular Biology & Microbiology, Faculty of Biological Science and Technology, University of Isfahan, Isfahan, P.O. Box: 8174673441, Iran

**Keywords:** Quercetin, Superparamagnetic iron oxide nanoparticles, Diabetes, SIRT1/p66Shc-mediated pathway, Hippocampus

## Abstract

**Background:**

Quercetin (QC) possesses a variety of health-promoting effects in pure and in conjugation with nanoparticles. Since the mRNA-SIRT1/p66Shc pathway and microRNAs (miRNAs) are implicated in the oxidative process, we aimed to compare the effects of QC and QC-conjugated superparamagnetic iron oxide nanoparticles (QCSPIONs) on this pathway.

**Methods:**

Through the use of the chemical coprecipitation technique (CPT), SPIONs were synthesized, coated with dextran, and conjugated with quercetin. Adult male Wistar rats were given intraperitoneal injections of streptozotocin to look for signs of type 1 diabetes (T1D). The animals were randomized into five groups: the control group got deionized water (DI), free QC solution (25 mg/kg), SPIONs (25 mg/kg), and QCSPIONs (25 mg/kg), and all groups received repeat doses administered orally over 35 days. Real-time quantitative PCR was used to assess the levels of miR-34a, let-7a-p5, SIRT1, p66Shc, CASP3, and PARP1 expression in the hippocampus of diabetic rats.

**Results:**

In silico investigations identified p66Shc, CASP3, and PARP1 as targets of let-7a-5p and miR-34a as possible regulators of SIRT1 genes. The outcomes demonstrated that diabetes elevated miR-34a, p66Shc, CASP3, and PARP1 and downregulated let-7a-5p and SIRT1 expression. In contrast to the diabetic group, QCSPIONs boosted let-7a-5p expression levels and consequently lowered p66Shc, CASP3, and PARP1 expression levels. QCSPIONs also reduced miR-34a expression, which led to an upsurge in SIRT1 expression.

**Conclusion:**

Our results suggest that QCSPIONs can regulate the SIRT1/p66Shc-mediated signaling pathway and can be considered a promising candidate for ameliorating the complications of diabetes.

## Background

Diabetes mellitus (DM), which is defined by hyperglycemic symptoms, is characterized by insulin resistance and insulin insufficiency [1]. Reactive oxygen species (ROS) damage lipids, proteins, and nucleic acids in persistent hyperglycemia, producing secondary oxidation products such as malondialdehyde (MDA) and 4-hydroxynonenal (4-HNE) [2]. Central nervous system (CNS) oxidative damage, which is linked to neuronal cell death and cognitive issues, is one potential consequence of diabetes [1]. Diabetes-related cognitive impairment involves several cellular stress-sensitive signaling pathways [3–5]. The SIRT1/p66Shc pathway and other associated genes, including CASP3, PARP1, miR-34a, and let-7a-5p, can be affected by oxidative stress on their mRNA expression.

One of the mammalian sirtuins encoded by SIRT1 is known as Sirtuin 1 (SIRT1), also known as NAD^+^-dependent lysine deacetylase Sirtuin 1. It has an essential function in cell survival, neurogenesis, and synaptic plasticity, and ameliorates diabetes-induced memory impairment [6, 7]. SIRT1 is found in the hippocampus, which plays a role in the cognitive process, as well as the adipose tissue, pancreas, skeletal muscle, liver, and brain [6]. Oxidative stress can prevent SIRT1 activity by decreasing its expression and NAD^+^ levels [8]. p66Shc is a pro-apoptotic protein encoded by the Shc1 gene locus and its expression is increased in diabetes.

This locus also produces 46- and 52-kDa proteins with one copy but two separate ATG start codons. These three isoforms share domains, but p66Shc also has an additional N-terminal collagen homology domain (CH2) [9, 10]. p66Shc is connected to the generation of ROS by the mitochondria, oxidative stress, and apoptosis induction [11]. By targeting the p66Shc promoter, SIRT1 can reduce the expression of p66Shc. Consequently, the SIRT1/p66Shc pathway is crucial for preventing the negative effects of oxidative stress [12]. In diabetes, p66Shc expression and activity can be increased by pathways such as amyloid-beta accumulation, protein kinase c (PKC) isoform activation, and SIRT1 impairment [13, 14].

These actions lead to the phosphorylation of p66Shc in the serine 36 domain of CH2. Activation of p66Shc can reduce FOXO expression and generate ROS in the plasma membrane through activation of RAC1 and NADPH oxidase [15]. After being phosphorylated in the cytosol, p66Shc can enter mitochondria and release cytochrome c into the cytosol. Together with APAF-1, it creates a complex that activates procaspase 9, which in turn activates downstream executive caspases such as caspase 3 (CASP3) and PARP1 cleavage [16–18]. CASP3 is an important effector protease, especially in neurodegenerative diseases, and its activation leads to brain tissue loss during apoptosis [19, 20]. The transfer of ADP-ribose units from NAD^+^ to nuclear target proteins, including PARP1 itself, is carried out by the PARP superfamily, which includes PARP1 [21]. Upon oxidative stress, massive DNA damage increases the activity of PARP1 and its expression for covalent PARylation and recruitment of DNA repair proteins to DNA damage sites. NAD^+^ Oxidation by PARP1 in response to DNA damage leads to ATP deficiency and subsequent cell death [21–23].

Noncoding microRNAs (miRNAs), approximately 22 nucleotides in length, are generally involved in physiological and pathological processes by inhibiting gene expression. Since each miRNA can regulate gene expression, disruption of this RNA can affect many biological processes [24]. These RNAs are involved in pancreatic organogenesis, β-cell differentiation, insulin secretion, and glucose homeostasis [25–27]. The results of a recent study showed a change in the expression of 316 miRNAs in an STZ-induced T1DM mouse model [28].

Recent studies reported the role of miR-34a in memory impairment, including diabetes-induced cognitive impairment [29, 30]. In this context, silencing of miR-34a ameliorated memory impairment by decreasing apoptosis in the hippocampus of diabetic mice [29]. Another miRNA candidate is the let-7 family, which is related to many cellular processes such as cell proliferation, tumor suppression, and stress response [31, 32]. Downregulation of let-7a-5p increases apoptosis of mesenchymal cells in patients with diabetic nephropathy [33]. Several studies have shown that let-7a-5p may target the genes p66Shc, CASP3, and PARP-1 [33–35]. Additionally, miR-34a may regulate SIRT1 [36–38]. Therefore, regulating the expression of miR-34a and let-7a-5p may be considered a therapeutic target to reduce the complications caused by DM.

A flavonoid polyphenol molecule called quercetin (QC) can be found in common foods like tea, fruits, seeds, and vegetables [39]. It has anti-cancer, anti-inflammatory, anti-diabetic, antioxidant, anti-allergic, anti-infective, cardioprotective, and neuroprotective effects [40]. QC reduces hyperglycemia and insulin resistance and increases glucose uptake and antioxidant enzyme activity [5]. This flavonoid exerts its protective effects against various diseases by regulating gene and miRNA expression [41]. Despite the many benefits of QC in medicine, its low solubility in aqueous media, low permeability, poor oral bioavailability, and biodegradation limit its applications [42].

Superparamagnetic iron oxide nanoparticles (SPIONs) are a group of paramagnetic nano-delivery systems with a diversity of diagnostic and biomedical applications [43] due to optimal physicochemical properties, including enhanced bioavailability, multifunctionality, colloidal stability, and tissue permeability [44, 45]. In recent years, we have obtained interesting results by applying QC-conjugated superparamagnetic iron oxide nanoparticles (QCSPIONs) in animal models and cell cultures [46, 47]. In diabetic rats, we demonstrated that oral administration of QCSPIONs (25 mg/kg) enhanced memory function [48]. The fundamental molecular pathways behind this process were investigated in further research. For instance, QCSPIONs have been shown to have anti-inflammatory and antioxidant benefits by regulating the NF-B/miR-146a and Nrf2/miR-27a signaling pathways in diabetic rats [49, 50]. We also highlighted how QCSPION affected the expression of genes involved in glucose metabolism and the miR-29 family as potential modulators in the hippocampus of diabetic rats [51].

We suggest that the deregulation of the SIRT1/p66Shc pathway and associated components may be a further mechanism behind ROS-induced memory impairment in the current investigation. In the previous studies of our group, the effect of QC, SPIONs and QCSPIONs on memory and learning of diabetic rats was investigated. In those studies, it was found that diabetes causes loss of memory and learning. Also, a series of parameters related to diabetes and QC’s influence on it were investigated. In addition, the expression of some genes were evaluated [48–51]. In the present study, we used hippocampus samples of our previous study. The aim of the current study is to investigate the effects of QCSPIONs on the expression levels of SIRT1, p66Shc, CASP3, PARP1, miR-34a, and let-7a-5p in compared with free QC in the hippocampus of diabetic rats to determine whether the stated pathway is engaged in this process.

## Methods

### Animals

In our previous study, 40 adult male Wistar rats weighing 200–230 g each were acquired from the Royan Institute (Isfahan, Iran). Rats were kept there for two months under controlled conditions (25 ±°C 2 °C temperature, 40–50% humidity). Throughout the experiment, all rats had unrestricted access to normal food and water. Regarding ethical considerations, the Guidelines for the Care and Use of Laboratory Animals (USA National Institute of Health Publication No. 80 − 23, updated 1996) were adhered to. The Isfahan University Animal Ethics Committee gave it their seal of approval (ethical approval code: IR.UI.REC.1400.066) [48].

### Experimental design for the induction of diabetes

Intraperitoneal injections of 20 mg/kg STZ were given for five days continuously to establish type one diabetes (Ebrahimpour Esmaeili et al. 2018). Blood sugar levels in all STZ rats were higher than 250 mg dL-1. The animals were distributed into five groups of eight at random: Normal control rats in Group 1 received deionized water (DI); diabetic rats in Group 2 received DI; diabetic rats in Group 3 received 25 mg/kg of free QC solution; diabetic rats in Group 4 received 25 mg/kg of Fe3O4 NPs; and diabetic rats in Group 5 received 25 mg/kg of QCFe3O4 NPs. Five days after the final STZ injection, all formulations were suspended in DI just before administration. They were then given at a dose every day for 35 straight days. Based on earlier research, the best QC dosage and duration for treating diabetes problems were chosen [52–54]. Rats were sacrificed after receiving injections of the anesthetics xylazine (10 mg/kg) and ketamine (100 mg/kg) at the final stage of the study. The hippocampal tissue was taken out and stored at -70 °C until needed. In the current study we did the experiment using the stored samples.

### Synthesis of QC conjugated Fe_3_O_4_ NPs (QCSPIONs)

We synthesized QCSPIONs as previously reported (Ebrahimpour Esmaeili et al. 2018). Using chemical coprecipitation (CPT), superparamagnetic iron oxide nanoparticles were made that were coated in dextran. Consequently, 200 mL of DI water was used to dissolve anhydrous FeCl3 (1.135 gr) and FeCl2 (0.695 gr). To create a solution with a pH of 9, ammonia solution was added to the mixture in the following step. 50 mL of water was used to dissolve 0.45 gr of dextran before adding it slowly to the mixture. At 90 °C, the resulting mixture was stirred for two hours. The dextran-coated superparamagnetic iron oxide nanoparticles were then harvested using a powerful external magnet. The resultant supernatant was then dried in an oven for an entire night at 70 °C after being rinsed with ethanol and deionized water. QC was combined with dextran-coated SPIONs and linked with EDC/NHS to create magnetic nanoparticles. The QCSPIONs were then washed with DI water and acetone, dried in a freeze-dryer, and recovered from suspension using an external magnet.

### Real-time quantitative PCR analysis

#### RNA extraction

To isolate RNA, hippocampal tissue (50 to 100 mg) was secluded from each sample and minced in a separate sterile Petri dish. According to the manufacturer’s protocol, total RNA was extracted from the samples using TRIzol solution (Invitrogen, Life Technologies, Grand Island, NY, USA). Homogenization of the sample was performed using a 2.5-cm³ syringe with a 21-g needle and repeated pipetting. The concentration and purity of the extracted RNA were determined using a Nanodrop spectrophotometer (Thermo Fisher Scientific, USA). To check the RNA quality in the 1% agarose gel, the presence of bands of ribosomal RNA (28 s, 18 s, 5.8 s) with minimal smear was considered. To exclude possible contamination with DNase and RNase, 1 μg of extracted RNA was treated with 1 U RNase-free DNase I (Thermo Fisher Scientific Inc, USA). All the above steps were performed under a laminar hood with gloves and RNase-free tubes under completely sterile conditions.

#### cDNA synthesis

For the synthesis of cDNA, a PrimeScript RT reagent kit from Takara (Japan) is employed. In a final volume of 10 μL, combine 500 ng RNA, 2 μL 5x PrimeScript buffer, 0.5 μL RT-enzyme, 0.5 μL oligo dT primer, and 0.5 μL random 6mer. Incubate for 15 min at 37 °C and 5 s at 85 °C.

Additionally, miRNA cDNA was created using the BON -miR miRNA 1st-strand kit (Bonyakhteh, Tehran, Iran, cat. no. BON209001). Since miRNAs don’t have a polyA tail at the 3’-UTR end, they were first extended in a polyadenylation process with a final volume of 20 μL at 37 °C for 30 min. After polyadenylation, the BON -miR miRNA 1st-strand cDNA Synthesis Kit carried out the cDNA synthesis procedure.

#### Real-time PCR

By means of 2X Master Mix Green, the cDNA synthesized in the preceding step was applied as a template for real-time PCR quantification of microRNA and mRNA (Ampliqon Odense, Denmark). To balance the amounts of mRNA expression, -actin was chosen as the reference gene. miR-U78 was a gene for miRNAs. These genes are useful reference genes for researching mRNA and miRNA expression because they exhibit steady expression levels under various cellular circumstances [49]. By means of the online OligoArchitect program (www.oligoarchitect.com/LoginServlet), primers for the genes under investigation were designed and then uploaded through blast to the NCBI website (www.NCBInlm.nih.gov/blast). To identify additional characteristics, the software OligoAnalyzer-1.0.3 was used. Bioneer provided the specified primers (City, Korea). Table 1 contains a list of the primer sequences utilized in this study. In a reaction with a final volume of 10 l, the mRNA expression levels were assessed using the qPCR technique. 5 μL of SYBR Green, 1 μL of cDNA, and 0.5 μL and 0.5 pM forward and reverse primers were combined in this reaction. Bonyakhteh Corporation developed and produced miRNA forward and universal reverse primers (Bonyakhteh, Tehran, Iran). The expression level of miRNAs was measured using a real-time PCR reaction. This 13 μL reaction had 6.5 μL of qPCR master mix, 0.5 μL of miRNA-specific forward primer, 0.5 μL of universal reverse primer, and one μL of cDNA. The following cycling conditions were used for real-time PCR tests: initial denaturation at 95 °C for 2 min, 40 cycles at 95 °C for 5 s each, and 60 °C for 30 s. An apparatus called chromo4 (Bio-Rad, USA) was used to conduct the reaction. The data was shown in the Opticon Monitor software once real-time PCR was completed. The software plotted and analyzed the amplification curve, threshold cycle, and melting curve for each gene in each sample. The specificity of the products was verified by examining the melting curves, and the mean threshold cycle (CT) was computed from repeated amplifications. Optimized PCR settings, primer concentrations, and cDNA concentrations were used. Using the ΔΔCt method, the degree of gene expression was compared between groups.

**Table 1:** Primers for Real-time PCR

Target bp	Forward primer 5ˊ → 3ˊ	Reverse primer 5ˊ → 3ˊ	Amplicon bp
β-actin	CTCTATGCCAACACAGTG	AGGAGGAGCAATGATCTT	123
SIRT1	CCAGTAGCACTAATTCCAA	CACCTAACCTATGACACAA	144
PARP1	CACAGTTATCGGCAGTAA	TCCAGTCTTCTCTTCGTA	90
p66Shc	CAGTGTTGTGGAATTATGTG	CAGAAAGCCTTCAGAGTAA	106
CASP3	ATTACGAAGCAGTGATGAT	TGGATTCAAGTTCTAAGACA	113

### Analytical statistics

Results were given as mean ± SEM and analyzed using the GraphPad Prism software tool to statistically research and create graphs (GraphPad Software, version 8.4.3 Inc., San Diego, CA, USA). When performing a one-way ANOVA and Tukey’s post hoc test for statistical analysis, *P* < 0.05 was considered a significant threshold.

## Results

### In silico analyses identify miR-34a as a potential SIRT1 regulator and let-7a-5p as a potential p66Shc, CASP3, and PARP1 regulator

**Fig. 1:**
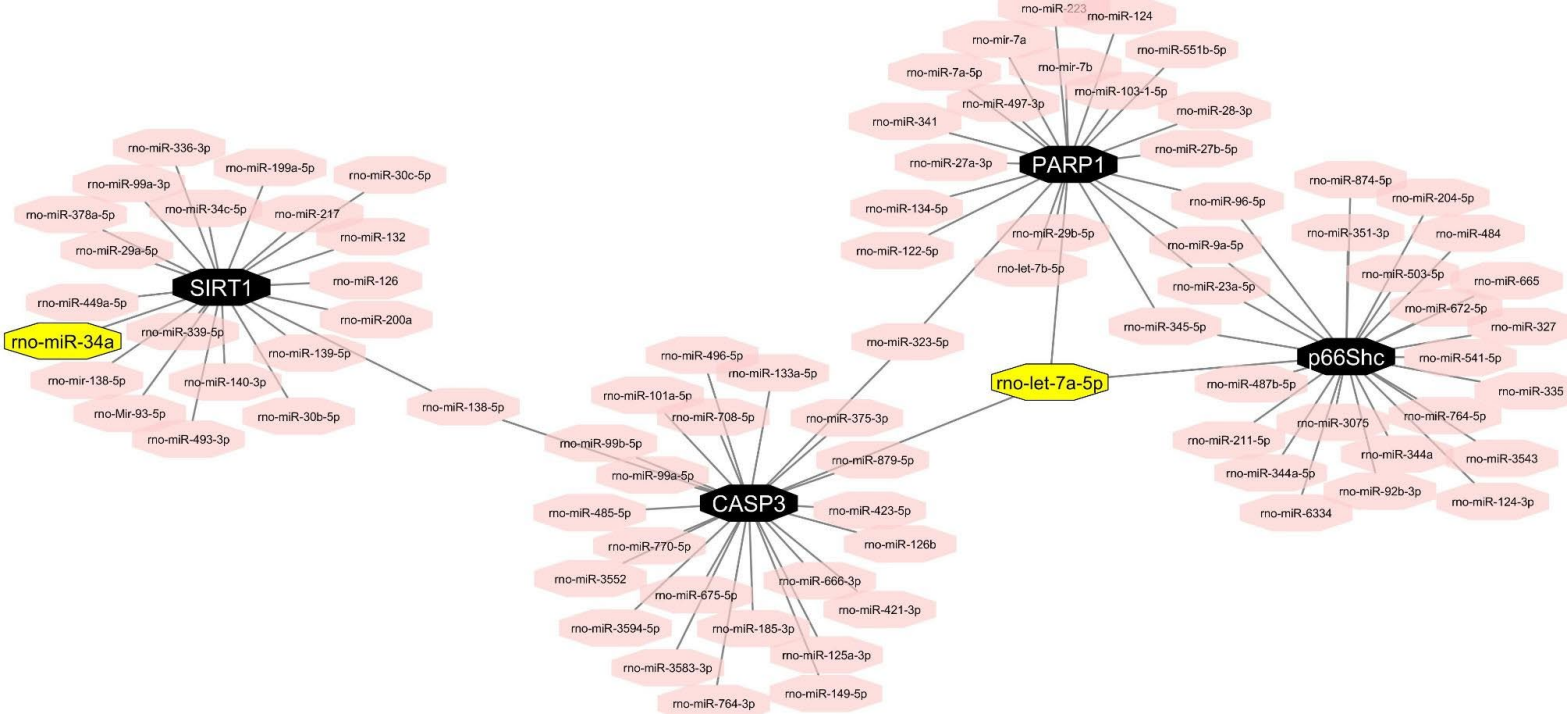
Potential regulation of four main genes in the SIRT1/p66Shc pathway. Among all miRNAs, let-7a-5p targets p66Shc, CASP3, and PARP1 commonly. SIRT1 is targeted by miR-34a. This network was drew by Mahnaz Karami using Cityscape (https://cytoscape.org/release_notes_3_8_0.html)

To identify miRNAs that target SIRT1 mRNA, we used databases that predicted and validated miRNA–target interaction and reviewed relevant previous studies. miRWalk 3 revealed hundreds of rat SIRT1-targeting miRNAs. In this database, several miRNAs were validated experimentally by luciferase assay, real-time PCR, western blot, and microarray methods based on the miRTarBase. Then, according to a comprehensive literature review [29, 38, 55], among the miRNAs involved in diabetes, especially in the hippocampus, we selected miR-34a, which is also present in the miRWalk 3 and has the validated diagnostic assay (http://mirwalk.umm.uni-heidelberg.de/rat/gene/309757/?page=9). We prepared a list of miRNAs that target p66Shc, CASP3, and PARP1 using miRWalk, miRDB, miRmap (https://mirmap.ezlab.org/), and miRTarBase (https://mirtarbase.cuhk.edu.cn/~miRTarBase/miRTarBase_2022/php/search.php) databases. Each miRNA in this list was checked in articles. Since let-7a-5p is expressed in the hippocampus and validated by methods, for instance, luciferase assay, western blot, and real-time PCR for targeting three considered genes, it was selected [33, 34, 56, 57] (Fig. 1).

### QC and QCSPIONs massively decrease the expression of miR-34a in the hippocampal region

**Fig. 2:**
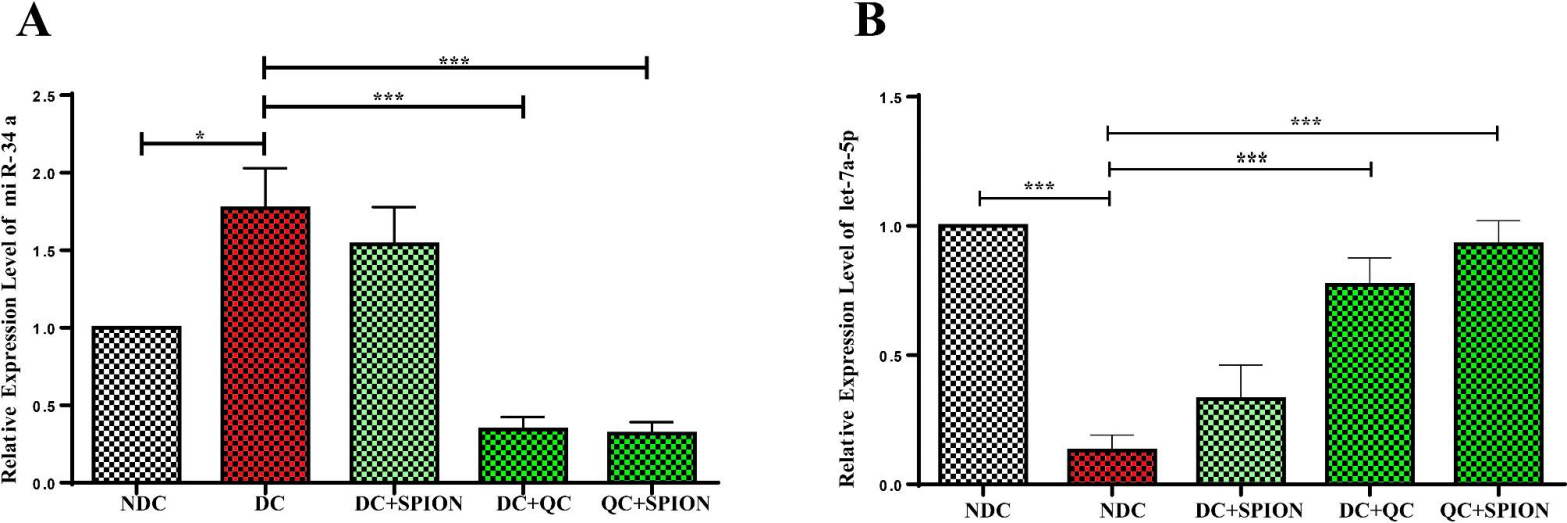
Effect of QC and QCSPIONs treatment on diabetic rats’ hippocampal miR-34a and let-7a-5p levels (**A**) and (**B**). NDC stands for non-diabetic control, DC for diabetic control, DC + SPION for diabetic treatment using a superparamagnetic iron oxide nanoparticle, DC + QC for diabetic treatment using quercetin, and DC + QCSPION for diabetic treatment using a quercetin-conjugated iron oxide nanoparticle.* *P* < 0.05 and *** *P* < 0.001 and *P* < 0.0001 versus DC group (one-way ANOVA, Tukey’s multiple comparison tests). Mean values are expressed together with the standard error of the mean (SEM) (n = 5 per group)

Figure 2A displays the expression levels of miR-34a in the rat hippocampus. When compared to the control group, the expression level of miR-34a in the diabetes group significantly increased (*p* < 0.05). When compared to the diabetic group, the rats fed with free QC had significantly lower levels of miR-34a expression (*p* < 0.001). Rats given QCSPION treatment also showed a similar amount of miR-34a (*p* < 0.001). Therefore, there was not significant difference between QC and QCSPIONs groups in the expression of miR-34a.

### QC and QCSPIONs significantly enhance let-7a-5p expression in the hippocampus

According to the results of real-time PCR, a significant decrease was observed in let-7a-5p levels of the diabetic group compared to the control group (*p* < 0.0001). In comparison to the diabetic group, treatment with free QC significantly increases let-7a-5p expression levels (*p* < 0.0001). Furthermore, QCSPION treatment increases let-7a-5p expression levels more than free QC (*p* < 0.0001) (Fig. 2B).

### The expression of SIRT1 mRNA in the hippocampus is considerably increased by QC and QCSPIONs

**Fig. 3:**
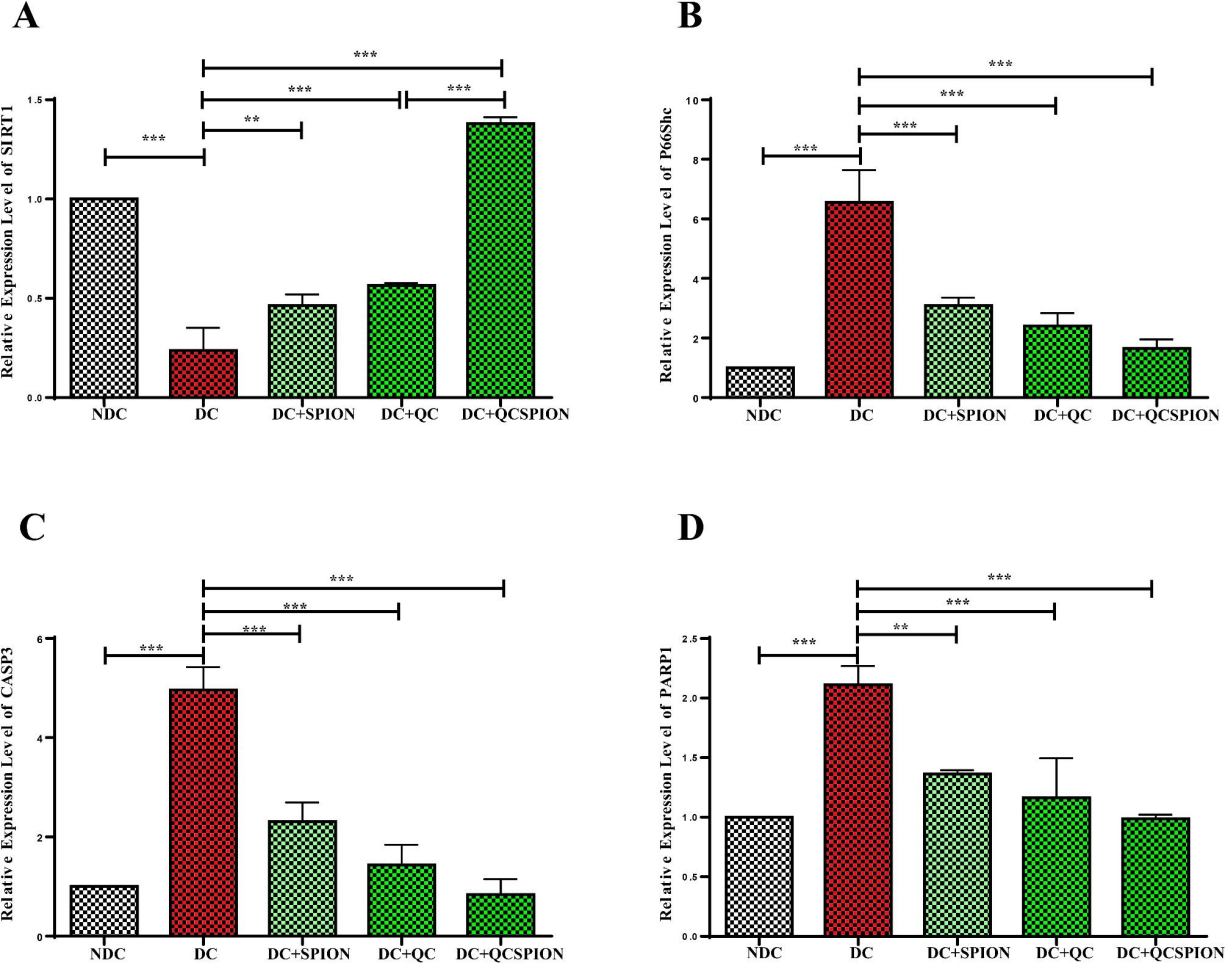
Effect of QC and QCSPIONs administration on (**A**) SIRT1, (**B**) p66Shc, (**C**) CASP3, and (**D**) PARP1 mRNA expression levels in the hippocampus of diabetic rats. NDC stands for non-diabetic control, DC for diabetic control, DC + SPION for diabetic treatment using a superparamagnetic iron oxide nanoparticle, DC + QC for diabetic treatment using quercetin, and DC + QCSPION for diabetic treatment using a quercetin-conjugated iron oxide nanoparticle. ** *P* < 0.01 and *** *P* < 0.001 and *P* < 0.0001 versus DC group (one-way ANOVA, Tukey’s multiple comparison tests). Mean values are expressed together with the standard error of the mean (SEM) (n = 5 per group)

Real-time PCR experiments revealed that ’the levels of SIRT1 expression in the diabetic rats’ hippocampus were considerably lower than those of the control group (*p* < 0.0001). As can be seen in Fig. 3A, SIRT1 expression was significantly higher in the group that had received QC treatment compared to the diabetic group. Interestingly, QCSPIONs could increase SIRT1 expression up to normal levels (*p* < 0.0001), which indicates the function of QCSPIONs in the regulation of SIRT1 mRNA expression levels more effectively than free QC. When Q‌C group is compared with QCSPIONs group, a significant difference in mRNA expression is observed (*p* < 0.0001).

### The hippocampus’s expression of p66Shc mRNA was reduced significantly by QC and QCSPIONs

In comparison to the control group, diabetic rats’ hippocampal p66Shc mRNA expression level significantly increased, according to real-time PCR results (*p* < 0.0001). When compared to the diabetic group, free QC dramatically reduced the expression of p66Shc in the treated group (*p* < 0.0001). As shown in Fig. 3B by QCSPIONs treatment, p66Shc expression levels decreased more than QC treatment but it was not significant.

### QC and QCSPIONs decrease CASP3 mRNA expression in the hippocampus

When compared to the control group of rats, the diabetic group’s hippocampus had considerably higher levels of CASP3 expression (*p* < 0.0001). In the group that had QC and QCSPIONs treatment, this up-regulation was reversed (*p* < 0.0001). (Fig. 3C). Comparison between QC group and QCSPION group did not show any significant difference.

### QC and QCSPIONs significantly decrease PARP1 mRNA expression in the hippocampus

Real-time PCR results showed that there was a significantly higher level of PARP1 mRNA expression in the diabetes group’s hippocampus as compared to the control group (*p* < 0.0001). When compared to the diabetic group, the rats given free QC showed significantly reduced levels of PARP1 mRNA (*p* < 0.001). Moreover, the amount of PARP1 mRNA was significantly lower in the QCSPION-treated group compared to the diabetic’s group (*p* < 0.0001) (Fig. 3D). As shown in the Fig. 3D there is no significant difference between QC group and QCSPIONs group.

## Discussion

We look for alternatives to chemical compounds made from herbs because using them to cure disorders can have side effects. Despite QC being a strong antioxidant, it cannot cross the BBB because of low solubility, stability, and tissue distribution [49]. Using iron oxide nanoparticles (IONPs) could overcome these limitations [46]. Nonetheless, several studies have documented the cytotoxicity of IONPs in brain tissue. Iron homeostasis is disturbed by iron ions produced from the core of nanoparticles and byproducts of IONP metabolism [58–60]. Also, iron accumulation in the neural tissue can aggravate neurodegenerative disorders through protein aggregation and oxidative stress [61]. However, modifying the Physico-chemical properties of IONPs and simultaneous use with bioactive compounds such as QC that have both antioxidant and chelating properties can be an appropriate solution to neutralize iron overload [62, 63]. Simultaneous use of QC in a conjugated form not only reduces the IONPs’ cytotoxicity but also increases the QC bioavailability [62]. In prior research, we found that QCSPIONs with lower QC concentrations than free QC could greatly enhance learning and memory in diabetic rats without neurotoxicity.

Our findings demonstrated that diabetes elevated miR-34a, which in turn decreased SIRT1’s expression levels as its target gene. In addition, let-7a-5p’s recognized target genes, p66Shc, CASP3, and PARP1, were elevated in diabetes circumstances whereas let-7a-5p was downregulated. These results are in line with those of earlier research. Recent investigations demonstrated SIRT1’s critical role in diabetic memory impairment [6]. This is because low levels of SIRT1 activity and expression, particularly in the hippocampus areas CA1, CA3, and dentate gyrus, cause inflammation, oxidative stress, and neuronal death, which impairs learning and memory [64, 65]. In STZ-induced diabetic rats, Du et al. found that SIRT1 activity diminished significantly. Moreover, reduced SIRT1 activity, NAD^+^ depletion, and enhanced ERK1/2 phosphorylation of tau protein were all associated with impaired cognitive function in rats [66]. Donmez et al. revealed the role of SIRT1 in the inhibition of the formation of Aβ aggregates [67]. According to previous knowledge of the interaction between SIRT1 and PARP1, which also share a common cofactor (NAD^+^), we evaluated the SIRT1 and PARP1 mRNA expression levels. PARP1 promoter is negatively regulated by the NAD^+^-dependent deacetylase activity of SIRT1. Decreased levels and activity of SIRT1 induced by oxidative stress cause PARP1 overexpression and lead to excessive NAD^+^ consumption by PARP1 [57]. On the other hand, ROS-induced massive DNA damage triggers PARP1-induced NAD^+^ depletion and cell death ( [57]. Activating inflammatory proteins such as iNOS and NF-ҡB is another destructive function of PARP1 [68]. Gisslen et al. revealed that hyperglycemic conditions lead to a 1.5-fold increase in PARP1 mRNA expression in the neonatal rat’s cerebral cortex [69].

It is reported that hyperglycemia by increasing fatty acids and through p66Shc enhances miR-34a expression and subsequently decreases SIRT1 expression. While inhibition of miR-34a or p66Shc prevents SIRT1 downregulation and oxidative stress [70]. According to Zhang et al., memory impairment in diabetic mice is caused by an enhancement in the expression of apoptosis-inducing genes and hippocampus miR-34a. Silencing of the miR-34a gene enhances mental performance and protects hippocampus neurogenesis [29]. These reports agree with our findings concerning the interaction between hippocampal SIRT1, PARP1, and miR-34a in diabetic rats.

As mentioned, upregulation of p66Shc and CASP3 in diabetes aggravates the complications of the disease. Camici et al. in 2007 reported an increase in p66Shc protein expression in the aorta of diabetic rats [71]. Minami et al. in 2018 observed that the p66Shc expression increased in the brain of type 1 and type 2 diabetic rats in comparison to non-diabetic rats. To consider the possible role of p66Shc in the brain of animal models, they generated p66Shc knockout gene diabetic mice. They found mutant mice more resistant to oxidative stress and generated less ROS in the brain. They also had less insulin resistance and significantly improved cognitive function (Minami Sonoda et al. 2018). Also, in a study accompanied in 2017 by Derungs et al., in PSAPP mice whose p66Shc was deleted, improved survival and reduced cognitive impairment have been observed [72]. Our data are also consistent with these studies. Real-time PCR results show that hyperglycemia has significantly enhanced the expression of p66Shc and CASP3 in the hippocampus of diabetic rats. So that the expression level of p66Shc and CASP3 in diabetic rats increased by 6.5 and 4.9 times respectively compared to the control group.

let-7a-5p as a microRNA that targets three of our desired genes: p66Shc, CASP3, and PARP1, was selected in our research. According to real-time PCR results, diabetes reduces let-7a-5p expression in the hippocampus of diabetic rats 7.7-fold less than in the control group, which is consistent with the outcomes of microarray and real-time PCR tests conducted in 2014 and 2015. Several investigations have demonstrated a reduction in the expression of let-7a-5p in the plasma of diabetic individuals and the pancreatic islets of mice with type 1 diabetes [73, 74].

**Fig. 4:**
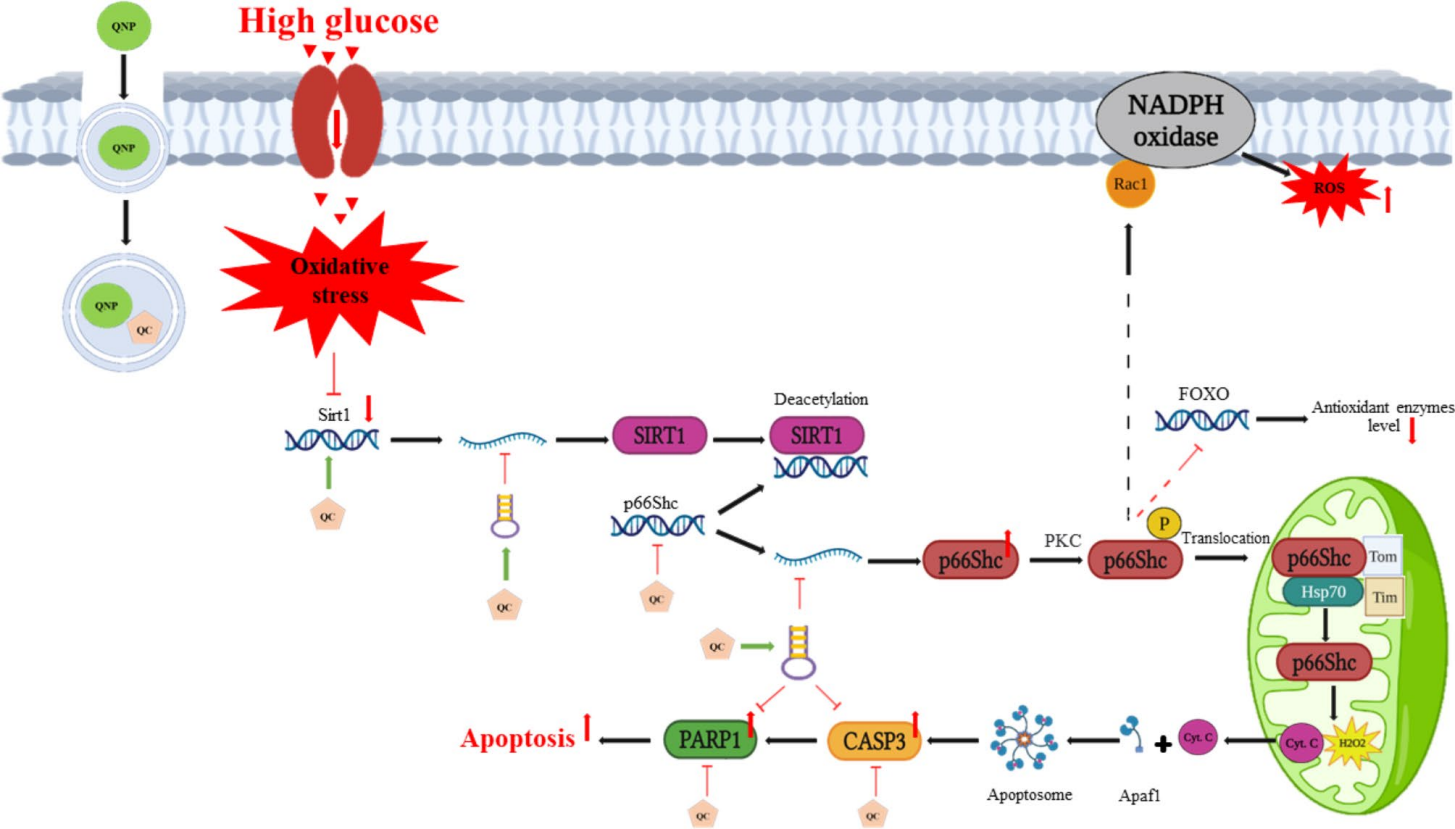
Schematic representation of the SIRT1/p66Shc pathway’s genes and miRNA affected by QCSPION’s antioxidant effects in the hippocampus of diabetic rats. Mahnaz Karami used BioRender and PowerPoint to create this figure

Our experiment revealed that QC, especially in conjugated form with SPIONs improved the expression of the SIRT1, p66Shc, CASP3, PARP1, miR-34a, and let-7a-5p to normal levels (Fig. 4). Peng et al. indicated that QC increased the activity and levels of hepatic SIRT1 protein and subsequent activation of Akt improved lipid and glucose metabolism in STZ-treated diabetic rats [75]. Sarubbo et al. reported that long-term treatment with QC in old rats has restorative effects on memory performance by increasing SIRT1 levels and decreasing NF-ҡB levels in the hippocampus [76]. Hu et al. reported that QC ameliorates diabetes-induced encephalopathy via SIRT1/ER stress pathway in db/db mice [77]. On the other hand, recent reports have confirmed the inhibitory effects of QC on PARP1. Boesten et al., through the exposure of endothelial cells to high glucose concentrations, observed a significant increase in the expression of PARP1 mRNA. Among the four flavonoids (QC, rutin, flavone, and sorbinil) only cells treated with QC showed a significant decrease in PARP1 expression to normal levels. In addition, QC and flavone increased NAD^+^ to favorable levels [78]. Recent reports about the involvement of miR-34a in the SIRT1/p66shc pathway, as well as the regulatory effects of flavonoids such as resveratrol and dihydromyricetin on miR-34a [12, 79, 80]. We investigated the effect of QC and QCSPIONs on miR-34a expression. Both free QC and its conjugated form equally normalized the downregulation of miR-34a. Thus, we can propose that QC like other previously investigated flavonoids directly regulates miR-34a. Moreover, it has been demonstrated that miR-34a overexpression inhibits SIRT1 post-transcriptional expression [12]. According to a 2007 study by Dihal, QC can lower the expression of Shc1 in rats with colon cancer [81]. In 2017, a study by Park et al. showed that QC could prevent nerve damage by inhibiting CASP3 and the apoptosis pathway in the brain [82]. Also, in the same year, Ola et al. determined that treatment with QC for diabetic rats reduces the expression level of CASP3 in the diabetic retina [83]. An enhancement in the expression of caspase was seen in the diabetic group in comparison to the control group in a recent investigation on the seminal vesicles of type 1 diabetic rats. Yet following QC therapy, a substantial drop in caspase expression was noted [84]. A lung tissue study in patients with lung cancer showed an increased expression of the let-7 family in a group of adenocarcinoma patients who smoked in the past and had a QC-rich diet, compared to the group that consumed less QC [85]. In another study, Appari and his colleagues concluded that QC with green tea catechin and sulforaphane induces let-7a in pancreatic ductal adenocarcinoma cells and prevents cancer progression [86].

## Conclusion

This study revealed that hyperglycemia-related oxidative stress can affect the molecules involved in the SIRT1/p66Shc-mediated pathway. According to the in silico analysis and previous studies, SIRT1 was recognized as the target gene of miR-34a, and p66Shc, CASP3, and PARP1 were identified as the target genes of let-7a-5p. The dysregulation of these microRNAs alongside their target genes leads to neuronal loss and cognitive dysfunction. Both free QC and QCSPIONs could control the expression level of mentioned genes. As the QCSPIONs-treated group received lower QC levels than the free QC-treated group it can be concluded QCSPIONs can be more effective than free QC in regulating SIRT1, p66Shc, CASP3, PARP1, miR-34a, and let-7a-5p expression levels in the diabetic rats hippocampus. Therefore, QC conjugated with superparamagnetic iron oxide nanoparticles can be considered a promising approach for improving diabetes-related cognitive impairment.

## Data Availability

The datasets used and/or analysed during the current study are available from the corresponding author on reasonable request.

## List of abbreviations

CASP3: Caspase 3
CNS: Central nervous system
CPT: Coprecipitation
DM: Diabetes mellitus
kDa: Kilodalton
miRNAs: MicroRNAs
PARP1: Poly (ADP-ribose) polymerase 1
PKC: Protein kinase C
QC: Quercetin
QCSPIONs: Quercetin-conjugated superparamagnetic iron oxide nanoparticles
qPCR: Quantitative polymerase chain reaction
ROS: Reactive oxygen species
Shc: Src homology and collagen
SIRT1: Sirtuin1
